# Preliminary Evaluation of Tick Protein Extracts and Recombinant Ferritin 2 as Anti-tick Vaccines Targeting *Ixodes ricinus* in Cattle

**DOI:** 10.3389/fphys.2018.01696

**Published:** 2018-12-05

**Authors:** Sarah Knorr, Juan Anguita, Julen T. Cortazar, Ondrej Hajdusek, Petr Kopáček, Jos J. Trentelman, Olivia Kershaw, Joppe W. Hovius, Ard M. Nijhof

**Affiliations:** ^1^Institute for Parasitology and Tropical Veterinary Medicine, Freie Universität Berlin, Berlin, Germany; ^2^Center for Cooperative Research in Biosciences (CIC bioGUNE), Derio, Spain; ^3^Ikerbasque, Basque Foundation for Science, Bilbao, Spain; ^4^Institute of Parasitology, Biology Centre, Czech Academy of Sciences, České Budějovice, Czechia; ^5^Center for Experimental and Molecular Medicine, Academic Medical Center, University of Amsterdam, Amsterdam, Netherlands; ^6^Institute of Veterinary Pathology, Freie Universität Berlin, Berlin, Germany

**Keywords:** *Ixodes ricinus*, anti-tick vaccine, salivary glands extract, midgut extract, ferritin, artificial tick feeding

## Abstract

Anti-tick vaccines have the potential to be an environmentally friendly and cost-effective option for tick control. In vaccine development, the identification of efficacious antigens forms the major bottleneck. In this study, the efficacy of immunization with recombinant ferritin 2 and native tick protein extracts (TPEs) against *Ixodes ricinus* infestations in calves was assessed in two immunization experiments. In the first experiment, each calf (*n* = 3) was immunized twice with recombinant ferritin 2 from *I. ricinus* (IrFER2), TPE consisting of soluble proteins from the internal organs of partially fed *I. ricinus* females, or adjuvant, respectively. In the second experiment, each calf (*n* = 4) was immunized with protein extracts from the midgut (ME) of partially fed females, the salivary glands (SGE) of partially fed females, a combination of ME and SGE, or adjuvant, respectively. Two weeks after the booster immunization, calves were challenged with 100 females and 200 nymphs. Blood was collected from the calves before the first and after the second immunization and fed to *I. ricinus* females and nymphs using an *in vitro* artificial tick feeding system. The two calves vaccinated with whole TPE and midgut extract (ME) showed hyperemia on tick bite sites 2 days post tick infestation and exudative blisters were observed in the ME-vaccinated animal, signs that were suggestive of a delayed type hypersensitivity (DTH) reaction. Significantly fewer ticks successfully fed on the three animals vaccinated with TPE, SGE, or ME. Adults fed on the TPE and ME vaccinated animals weighed significantly less. Tick feeding on the IrFER2 vaccinated calf was not impaired. The *in vitro* feeding of serum or fresh whole blood collected from the vaccinated animals did not significantly affect tick feeding success. Immunization with native *I. ricinus* TPEs thus conferred a strong immune response in calves and significantly reduced the feeding success of both nymphs and adults. *In vitro* feeding of serum or blood collected from vaccinated animals to ticks did not affect tick feeding, indicating that antibodies alone were not responsible for the observed vaccine immunity.

## Introduction

*Ixodes ricinus* is a tick species which is widespread in Europe and can transmit various bacterial, protozoal and viral pathogens of medical and veterinary importance, including the causal agents of Lyme borreliosis, tick-borne encephalitis (TBE) virus and babesiosis. Multiple studies have shown that the incidence of both Lyme borreliosis and TBE in several European countries have increased over the last decades (Smith and Takkinen, 2006; Fulop and Poggensee, 2008; Sykes and Makiello, 2017; Radzisauskiene et al., 2018). Lyme borreliosis is also the most common zoonotic vector-borne pathogen in the United States where *I. scapularis*, a sister species of *I. ricinus*, is the main arthropod vector (Schwartz et al., 2017).

The abundance and activity of *I. ricinus* ticks depends on abiotic factors, including relative humidity and temperature, as well as biotic factors such as adequate vegetation cover and vertebrate host availability (Randolph et al., 2002). *I. ricinus* is a three-host tick species with a broad host range; larvae and nymphs feed predominantly on rodents and birds, whereas the key reproduction hosts for adults are larger mammals (Gray et al., 1998). Control of *I. ricinus* and associated tick-borne diseases include personal preventive measures, such as the avoidance of tick habitats and a prompt removal of attached ticks, as well as environmental-based approaches, including habitat modification, a reduction of host densities or treatment of wildlife hosts with acaricides (Pound et al., 2012; Sprong et al., 2018). Another alternative targeted at controlling the tick vector is the use of anti-tick vaccines that would interfere with tick feeding and survival or pathogen transmission (Parizi et al., 2012; Sprong et al., 2014).

The first observations that animals repeatedly infested with ticks can develop an immune response that results in the rejection of ticks and that injection of tick extracts may also result in a partial immunity were made by William Trager in the 1930s (Trager, 1939a,b). This and similar studies formed the foundation for work by Australian scientists which led to the identification of the Bm86 antigen. This antigen is the principal component of the only commercialized anti-tick vaccine targeting an ectoparasite, the one-host tick *Rhipicephalus microplus*, to date (reviewed in Willadsen, 2004). The Bm86 protein was identified following multiple cycles of biochemical fractionation of immunogenic tick midgut extracts (MEs) followed by immunization trials with parasite challenges, with increasingly simpler protein mixtures being used for immunization in each successive cycle (Willadsen et al., 1989). Immunization with recombinant Bm86 was subsequently shown to be effective against *R. microplus* and a number of other tick species and homologs of Bm86 were identified in all main ixodid tick genera (de Vos et al., 2001; Nijhof et al., 2007). Immunization with two Bm86 orthologs isolated from *I. ricinus* was, however, not effective against conspecific tick infestations in rabbits (Coumou et al., 2015). More promising results for *I. ricinus* were obtained by immunization of rabbits with recombinant ferritin 2 (FER2). This protein is secreted by the tick midgut into the hemocoel and acts as an iron transporter, thus playing a pivotal role in the iron metabolism of ticks (Hajdusek et al., 2009). Immunization with recombinant FER2 in rabbits resulted in a reduction in tick numbers, engorgement weight and fertility rate of *I. ricinus* females feeding on immunized animals. Similar effects were observed for *R. annulatus* and *R. microplus* ticks feeding on cattle immunized with recombinant *R. microplus* FER2 (RmFER2) (Hajdusek et al., 2010). Other recombinant proteins that were evaluated for their efficacy in controlling *Ixodes* infestations in rabbits, mice and guinea pigs include subolesin (Almazan et al., 2005), tick cement protein 64TRP (Trimnell et al., 2005), the elastase inhibitor Iris (Prevot et al., 2007), sialostatin L2 (Kotsyfakis et al., 2008), a putative metalloprotease (Metis 1) (Decrem et al., 2008), anti-complement proteins IRAC I and IRAC II (Gillet et al., 2009), a cyclin-dependent kinase (Gomes et al., 2015), and aquaporin (Contreras and de la Fuente, 2017).

In this pilot study, we aimed to investigate if immunization of cattle to reduce *I. ricinus* tick feeding and reproduction is possible. To this purpose, we evaluated the use of recombinant FER2 as well as native tissue extracts. The cow was chosen as an animal model since it is a suitable host for infestations with high numbers of *I. ricinus* and also because large blood volumes can be safely drawn without affecting the animal’s health (Wolfensohn and Lloyd, 2013). This facilitated the subsequent evaluation of collected antisera within an artificial tick feeding system (ATFS) to study individual components of the immunological response on tick feeding (Contreras et al., 2017).

## Materials and Methods

### Calves and Ticks

Six-month-old Holstein-Friesian calves were purchased from a local dairy farm. Calves were housed on pastures of the Institute for Parasitology and Tropical Veterinary Medicine in Berlin during the first 7 weeks of the study (d0–d49). The pastures were considered to be free of ticks; natural tick infestations were not observed during this period. One week prior to tick challenge, they were moved to an enclosed cattle pen for the duration of the tick infestation. *I. ricinus* ticks for the *in vivo* challenge originated from the tick breeding unit of the Institute for Parasitology and Tropical Veterinary Medicine of the Freie Universität Berlin. For the *in vitro* feeding experiments, half of the adult *I. ricinus* ticks originated from the tick breeding unit. Prior to use, these were mixed with an equal number of adult *I. ricinus* ticks collected from the vegetation in and around Berlin, as previous (unpublished) work suggested that this might improve the attachment rate of ticks in the ATFS. All animal experiments were conducted with approval of the commission for animal experiments (LAGeSo, Berlin, registration number G0210/15).

### Vaccine Preparation

#### Native Tick Protein Extracts

For the preparation of native tick protein extracts (TPEs), female *I. ricinus* ticks were prefer for 3–5 days on rabbits. Partially fed females weighing ∼30 to 70 mg were manually detached and washed for 30 s in 70% ethanol. They were subsequently dissected on a glass slide under ice-cold phosphate buffered saline (PBS, pH 7.2). The internal organs (midguts, salivary glands, Malpighian tubules, trachea, synganglia, and ovaries), were dissected and stored in PBS on ice. For preparation of the TPE used in the first immunization study, all internal organs except the midguts were pooled together. For the second immunization study, only the salivary glands and midguts were used for preparation of the salivary gland extract (SGE) and ME, respectively. Tissues were homogenized in an ultrasound homogenizer (Hielscher, UP100H) followed by centrifugation at 15,000 *g* for 30 min at 4°C. The supernatant of each extract was sterile filtered (0.2 μm non-pyrogenic filters, Sarstedt Germany) and stored at -20°C until use. Protein concentrations were measured by the CB-X^TM^ Protein Assay (G Biosciences, United States) according to the manufacturer’s instructions.

#### Ferritin 2 (FER 2)

Recombinant protein ferritin 2 from *I. ricinus* (IrFER2) was expressed in *E. coli* strain BL21 as previously described (Hajdusek et al., 2010).

Prior to immunization, TPE, SGE, and ME or recombinant IrFER2 were emulsified in a homogenizer (IKA Turrax T25 Mixer) with 1.5 mg saponin in 1 mL of Montanide ISA V50 adjuvant (SEPPIC, France) as specified by the adjuvant’s manufacturer.

### Study Outline

All calves were vaccinated intramuscularly (i.m.) twice at an interval of 6 weeks. Two weeks after the second immunization, each calf was challenged with ticks. The first study was performed with three calves: one calf received 100 μg IrFER2, the second calf ∼12 mg TPE (4 ME: 1 other internal organs), and the third calf was vaccinated with Montanide ISA 50 adjuvant and saponin only. The second study was performed with four calves: one calf was vaccinated with ∼6 mg SGE, one with ∼9 mg ME, one with a ∼8 mg combination of ME and SGE (4 ME : 1 SGE) and a control calf with Montanide ISA 50 adjuvant and saponin only.

### Tick Challenge

Two weeks after the second immunization, each animal was infested with 200 nymphs, 100 adult males, and 100 *I. ricinus* females. The ticks were equally divided over two linen bags, which were attached to the basis of the unshaved ears using adhesive tape (Leukoplast, BSN medical, Hamburg, Germany).

Following a resting period of 2 days to allow ticks to attach undisturbed, bags were checked twice per day and engorged nymphs or females were removed and weighed. Adult females were subsequently stored individually and nymphs were stored in small batches in glass tubes. The tubes containing the ticks were stored in a desiccator with 80% relative humidity (RH), which was placed in a climate chamber at 20°C and a light-dark-cycle of 14–10 h.

### *In vitro* Tick Feeding

The ATFS used for the feeding of plasma or whole blood from the vaccinated calves to *I. ricinus* ticks *in vitro* was based on a previously published protocol (Krober and Guerin, 2007) which was further optimized in house (Krull et al., 2017). In short, tick feeding units were made of borosilicate glass tubes with a 28 mm inner diameter, 2 mm wall thickness, and height of 65 mm. The feeding units were closed on one end with a silicone membrane with a thickness of 80–120 μm for adults and 70–90 μm for nymphs. The silicone membranes were attached to the tick feeding unit using silicone glue. A square piece of glass fiber mosquito netting, approximately 20 mm × 20 mm in size, was glued to the silicone membrane inside the feeding units used for adult ticks to provide tactile stimuli. Silicone glue was applied to two sides of the square mosquito netting only, leaving sufficient space for ticks to crawl underneath the netting. Bovine hair extract (Krull et al., 2017) and bovine hair were dispersed over the silicone membrane to stimulate tick attachment. Ten females with 5–7 males, or 50 nymphs were placed in each feeding unit, which was subsequently placed in a climate chamber set at 20°C, 80% RH, 5% CO_2_ and 14 h light–10 h dark cycle.

Before the first (d0) and 2 weeks after the second immunization (d56), 1.5 L blood was collected from each calf. Blood was centrifuged at 3,500 *g* for 30 min at 4°C to separate blood cells and plasma. Plasma was collected and stored at -20°C until use.

For the first study and nymphal tick feeding of the second study, collected plasma was mixed with the erythrocyte and buffy coat layer of bovine blood collected at a local slaughterhouse. Blood cells were washed twice and subsequently stored at 4°C in modified Vega y Martinez phosphate-buffered saline solution containing 20% glucose (Zweygarth et al., 1995). Blood cells were overlaid with two-thirds of buffer. Blood cells and stored plasma were mixed in a 2:1 ratio, resulting in whole blood with a packed cell volume (PCV) of 33%. Ticks were subsequently fed using the ATFS. Before each feeding, gentamycin (10 mg/mL) and 0.1 M adenosine triphosphate (ATP) were added to the blood meal.

During the second study, blood for the *in vitro* feeding of *I. ricinus* adults was collected from each calf on day 55, day 62, and day 68 in the presence of heparin (20 I.U./mL) and glucose (2 g/L). Prior to *in vitro* feeding, it was supplemented with gentamycin (10 mg/mL) and 0.1 M ATP and preheated to 37°C on a hot plate (Hot Plate 062, Labotect, Göttingen, Germany). Blood was changed twice per day. Detached and replete adults and nymphs were weighed and stored individually in a desiccator with 80% RH at RT.

### Enzyme-Linked Immunosorbent Assay (ELISA)

Blood was collected weekly from each calf in a serum tube (Microvette^®^, Sarstedt, Germany). After coagulation, blood samples were centrifuged at 2,800 *g* for 5 min. The serum was subsequently withdrawn and stored until use at -20°C. A 96-well plate (F-bottom, Medium binding; Greiner Bio-one) was coated with 2.5 μg/mL TPE, ME or SGE or 0.5 μg/mL IrFER2 in coating buffer (0.05 M carbonate-bicarbonate; pH 9.6) at 20°C for 1 h and at 4°C overnight. Serum samples diluted 1:200 in 0.05% PBS-Tween were added to each well and the plate was incubated at 37°C for 30 min. Mouse anti-bovine IgG antibody, horseradish peroxidase-conjugated (Acris Antibodies GmbH) was diluted 1:5000 in 0.05% PBS-T and incubated at 37°C for 30 min. O-phenylenediamine (OPD-P9187, Sigma Aldrich) was used as substrate and resulting color was measured with Epoch Microplate Spectrophotometer (BioTek) at 492 nm. The plate was washed three times with 0.05% PBS-Tween after each incubation step.

IgM, IgG1, and IgG2 antibody titration experiments were performed using eight serial dilutions, from 1:100 to 1:12,800, of sera from the first and second immunization experiment. Here, the amount of total protein used for coating was 0.5 μg/mL in carbonate buffer. The plates were then washed, blocked with 1% fetal bovine serum in PBS, incubated with the sera dilutions followed by specific secondary antibodies against bovine IgG1, IgG2, and IgM conjugated with horseradish peroxidase (Bio-Rad). The ELISAs were developed using HRP substrate (Kirkegaard and Perry Laboratories, Gaithersburg, MD, United States) and read at 450 nm.

### Western Blot

Five micrograms of purified Ferritin 2 or tissue extracts were resolved in 4–20% precast polyacrylamide gels (Bio-Rad) and transferred to nitrocellulose membranes (Bio-Rad). The membranes were blocked for 2 h in PBS + 5% skim milk, followed by incubation with calf sera in PBS containing 0.1% Tween 20 (PBST) at 1:1000 dilution for 2 h. The membranes were washed three times with PBST, and incubated with a 1:10,000 dilution of anti-bovine IgG (Life Technologies) conjugated with horseradish peroxidase for a period of 1 h. After extensive washing the membranes were briefly incubated with HRP substrate (SuperSignal West Femto Maximum Sensitivity Substrate, Life Technologies) and developed in a ChemiDoc Gel Imaging System (Bio-Rad).

### Skin Sample Collection and Histological Processing

Biopsies were taken from euthanized animals of the second study at d68 from areas where ticks had fed and processed using routine histological techniques.

### Statistical Analyses

Engorgement weight of adult ticks and nymphs were analyzed using GraphPad Prism version 5.03 for Windows, GraphPad Software, La Jolla, CA, United States^1^. For normally distributed data one-way ANOVA followed by Bonferroni’s multiple comparison test was performed. Kruskal-Wallis test and Dunn’s post-test was conducted for data with non-Gaussian distribution. *P* ≤ 0.05 was considered significant for all tests. *P*-values for number of ticks and molting rate of nymphs were calculated with mid-p-exact test using OpenEpi version 3.01^2^. *P*-values were adjusted according to Holm correction in RStudio version 3.4.3.

## Results

### Immune Response

**FIGURE 1:**
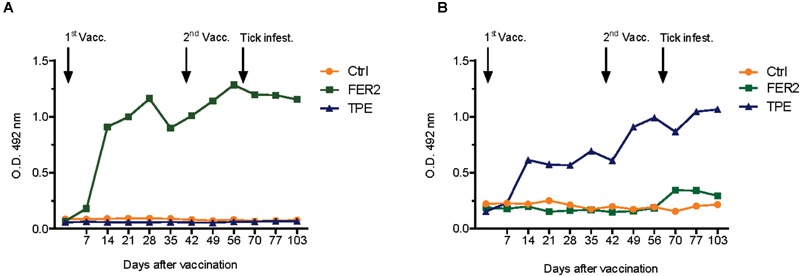
Antibody response in vaccinated animals of the first cattle immunization study. Bovine serum (diluted 1:200) antibody titers to **(A)** recombinant ferritin 2 (FER2) and **(B)** tick protein extract (TPE) were determined by indirect ELISA in cattle vaccinated with FER2, TPE, and adjuvant control.

**FIGURE 2:**
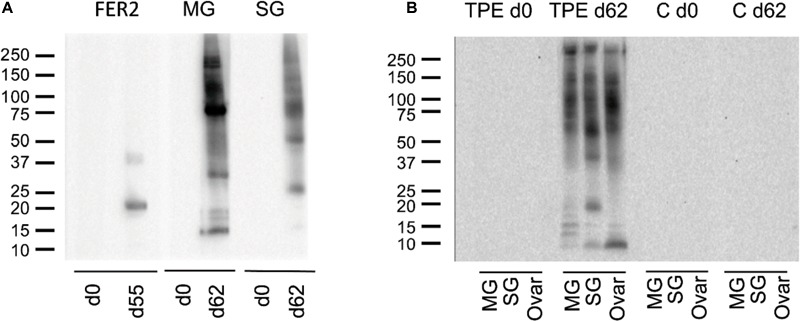
**(A)** Western blot of recombinant ferritin 2 (FER2), native midgut (MG), and salivary gland (SG) extracts, probed with pre-immune and immune sera of calves vaccinated with recombinant ferritin 2 (lanes 1 and 2) and tick protein extract (lanes 3 to 6); **(B)** western blot of native MG, SG, and Ovaries (Ovar) extracts, probed with pre-immune and immune sera of the calf vaccinated with tick protein extracts (TPE) and the control calf (C).

Indirect ELISA-results demonstrated that the IrFER2 and TPE vaccinated calf each developed antibody titers against recombinant IrFER2 and native TPE, respectively. Serum from the TPE vaccinated animal did not clearly recognize IrFER2 (Figure 1). These results were confirmed by Western blot, which also showed that immune sera from the TPE-immunized animal recognized ME, SGE and ovary proteins. Recombinant IrFER2 and native tick proteins were not recognized by pre-immune sera of the IrFER2 and TPE-immunized animals, respectively (Figure 2).

**FIGURE 3:**
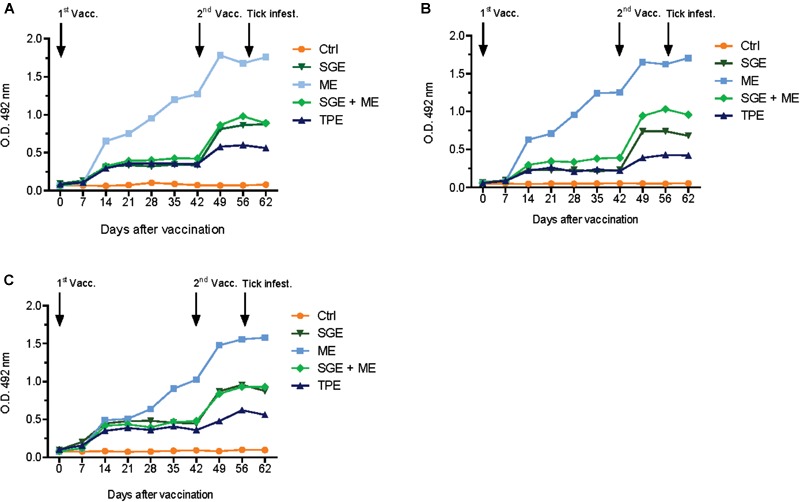
Antibody response in vaccinated animals of the second cattle immunization study. Bovine serum (diluted 1:200) antibody titers to **(A)** midgut extract (ME), **(B)** salivary gland extract (SGE), and **(C)** SGE and ME were determined by indirect ELISA in cattle vaccinated with ME, SGE and a combination of ME and SGE and the adjuvant control. For comparison purposes, serum from the TPE-vaccinated animal from the first immunization study was also included in the ELISA.

**FIGURE 4:**
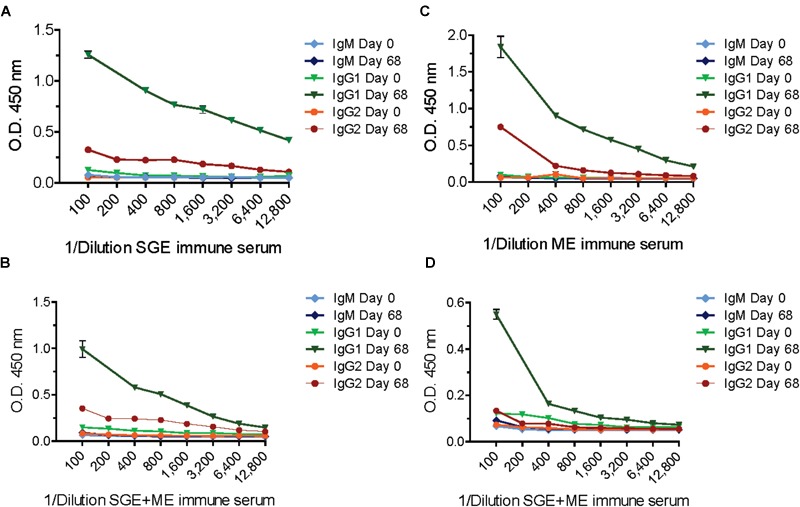
Titrated IgM, IgG1, and IgG2 antibody response in animals vaccinated with salivary gland extract (SGE), midgut extract (ME) or a combination of both SGE and ME (SGE+ME). Plates were coated with 0.5 μg/mL SGE **(A,B)** or ME **(C,D)**, respectively.

Calves from the second study, immunized with ME, SGE or a combination thereof developed higher antibody titers compared to the TPE-vaccinated calf from the first study. The calf immunized with ME showed a strong immune response against both ME and SGE and developed the highest antibody titer of all vaccinated calves (Figure 3). Antibody titration experiments predominantly showed a IgG1 and, to a lesser extent, IgG2 response in sera from the ME and SGE-immunized calves and the calf immunized with a combination of ME and SGE. A clear IgM response was not detected (Figure 4).

### Tick Challenge

**FIGURE 5:**
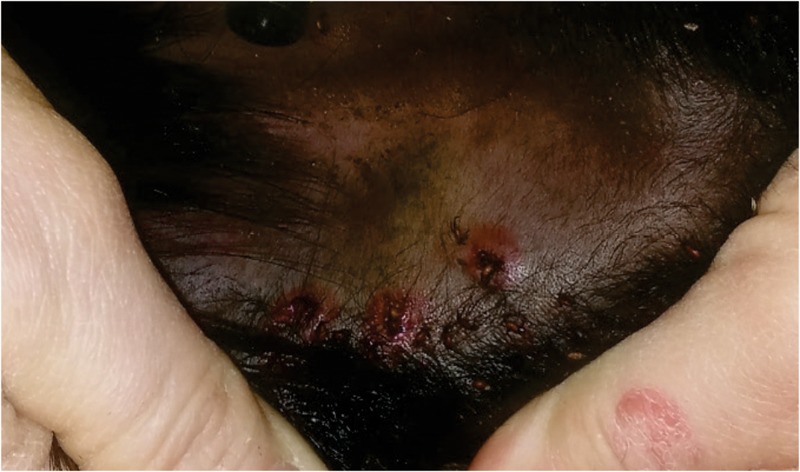
Photograph of the ear from the TPE vaccinated cow, 2 days after tick infestation, showing hyperaemia and oedema around the tick feeding sites.

Two days post tick infestation, hyperemia and oedema on the tick bite sites on the ears of TPE vaccinated calf were observed (Figure 5). These reactions were not observed on the ears of the IrFER2 vaccinated or control calf. On the negative control, 70 female ticks engorged, with a mean weight of 245.8 ± 85.3 mg. This was not statistically different from the IrFER2-vaccinated animal, on which 87 ticks engorged with a mean weight of with 268.6 ± 82.0 mg. Only 22 ticks fed to repletion on the TPE-immunized calf, with a significantly reduced engorgement weight of 126.6 ± 86.9 mg (*p* ≤ 0.001). The results for the nymphs fed on the TPE-vaccinated calf showed a similar trend: 14 from 200 nymphs were able to engorge compared to 128/200 and 145/200 nymphs from the control and IrFER2-vaccinated animal, respectively, but there was no significant difference between the engorgement weights (Table 1).

**Table 1:** Tick challenge and *in vitro* tick feeding results of 1st study.

Study	Life stage		Ctrl	TPE	IrFER2	TPE d0
Tick challenge	Adults	No	70^ab^	22^ac^	87^bc^	
		EW (mg)	245.8 ± 85.3^d^	126.6 ± 86.9^de^	268.6 ± 82.0^e^	
	Nymphs	No	128^f^	14^fg^	145^g^	
		EW (mg)	3.6 ± 0.9	3.1 ± 1.0	3.5 ± 0.9	
		Molting rate (%)	70^h^	36^hi^	71^i^	
		Sex of nymphs	54♀ 35♂	2♀ 3♂	59♀ 44♂	
*In vitro*	Adults	No	39	33	44	40
		EW (mg)	193.8 ± 74.8	177.8 ± 71.9	190.9 ± 79.3	201.4 ± 74.8
	Nymphs	No	84	79	70	
		EW (mg)	2.6 ± 0.9	2.7 ± 0.9	2.6 ± 0.8	
		Molting rate (%)	45^j^	49^k^	9^jk^	
		Sex of nymphs	16♀ 20♂	27♀ 17♂	2♀ 4♂	

*No, number of engorged ticks; EW, engorgement weight. Mean values ± SD tested in a one-way analysis of variance (ANOVA) followed by Kruskal-Wallis test for multi-group comparisons. *P*-values for number of ticks and molting rate (in percent) were tested by mid-p-exact test. *P*-values were adjusted according to Holm correction. Significantly differences are indicated by letters and *p*-values. The sex of nymphs was determined after molting.*

*^*a*^Mid-P-exact test, *p*-value adjustment by Holm correction; *p* < 0.0001. ^*b*^Mid-P-exact test, *p*-value adjustment by Holm correction; *P* = 0.0036. ^*c*^Mid-P-exact test, *p*-value adjustment by Holm correction; *p* < 0.0001. ^*d*^Kruskal-Wallis test, Dunn’s *post hoc*-test; *p* < 0.0001. ^*e*^Kruskal-Wallis test, Dunn’s *post hoc*-test; *p* < 0.0001. ^*f*^Mid-P-exact test, *p*-value adjustment by Holm correction; *p* < 0.0001. ^*g*^Mid-P-exact test, *p*-value adjustment by Holm correction; *p* < 0.0001. ^*h*^Mid-P-exact test, *p*-value adjustment by Holm correction; *P* = 0.0355. ^*i*^Mid-P-exact test, *p*-value adjustment by Holm correction; *P* = 0.0355. ^*j*^Mid-P-exact test, *p*-value adjustment by Holm correction; *p* < 0.0001. ^*k*^Mid-P-exact test, *p*-value adjustment by Holm correction; *p* < 0.0001.*

**FIGURE 6:**
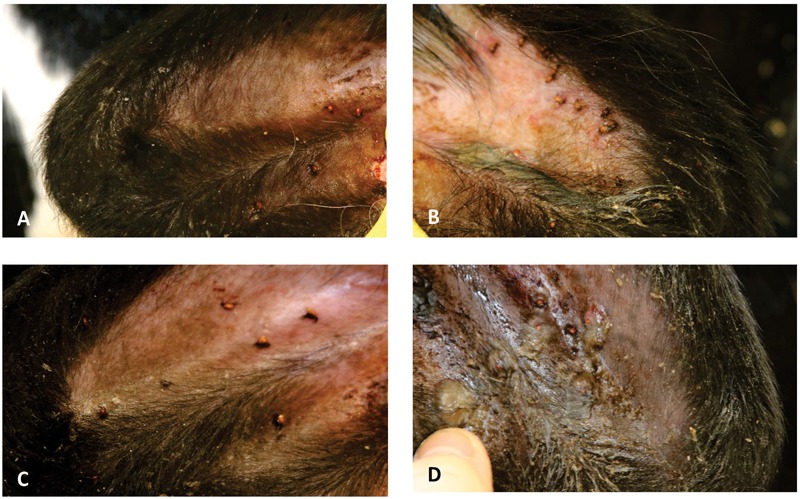
Photographs of the ears from the vaccinated calves of the second immunization experiment, 2 days post-infestation. **(A)** Control animal; **(B)** SGE+ME-immunized calf; **(C)** SGE-immunized calf; **(D)** ME-immunized calf.

**FIGURE 7:**
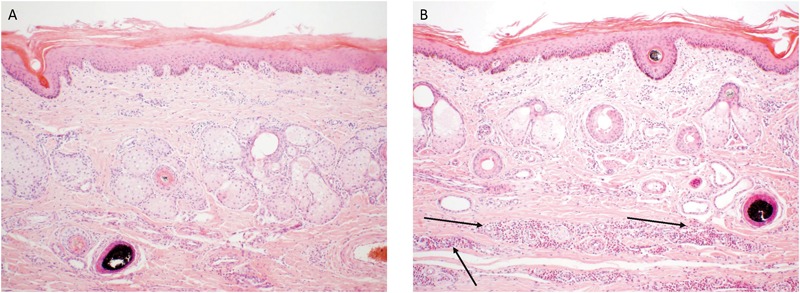
Histopathology of the ear skin from the control **(A)** and ME-immunized animal **(B)**. The arrows indicate the mixed inflammatory infiltrate mainly composed of eosinophils and macrophages in the dermis of the ME-immunized animal. The biopsies were taken at d68 post immunization and stained with hematoxylin and eosin (100× magnification).

Control and SGE vaccinated calves of the second study did not show a clear skin response at 2 days post tick infestation. The calf immunized with a combination of SGE and ME showed similar inflammatory signs on the tick bite sites with hyperemia as observed in the first study. The ME vaccinated animal showed a severe cutaneous reaction with a papular reaction at the tick attachment site with serous exudation 2 days post infestation (Figure 6). Histological examination of the ears could only be performed at d68 post immunization after the tick infestation in the second immunization experiment. It showed an extensive infiltration of the dermis with eosinophils and macrophages in the ears of animals immunized with SGE, ME and a combination of both extracts that was absent in the skin of the control animal (Figure 7).

Adults fed on SGE (187.8 ± 102.4 mg, *n* = 63) or SGE and ME (140.6 ± 89.5 mg, *n* = 25) immunized calves had a significantly (*p* ≤ 0.001) lower engorgement weight compared to adults fed on control (262.2 ± 70.8 mg, *n* = 69). Only two adult ticks were able to engorge on the ME vaccinated calf, with a significantly lower engorgement weight (79.0 ± 19.7 mg) in comparison to the control (*p* ≤ 0.05). For all vaccinated calves, a reduced number (SGE *n* = 23; ME *n* = 11; SGE and ME *n* = 20) of nymphs could engorge compared to the Ctrl (*n* = 92), but no significant differences between engorgement weights were observed. Mean engorgement weights and statistical results for adult ticks and nymphs of the second study are shown in Table 2.

**Table 2:** Tick challenge and *in vitro* tick feeding results of 2nd study.

Study	Life stage		Ctrl	SGE+ME	ME	SGE
Tick challenge	Adults	No	67^ab^	25^acd^	2^bce^	63^de^
		EW (mg)	262.1 ± 70.8^fg^	140.6 ± 89.5^f^	79.0 ± 19.7	187.8 ± 102.4^g^
	Nymphs	No	92^hij^	20^h^	11^i^	23^j^
		EW (mg)	3.5 ± 1.0	3.2 ± 1.0	3.6 ± 0.7	3.8 ± 1.1
		Molting rate (%)	86^k^	15^kl^	55^m^	100^lm^
		Sex of nymphs	42♀ 37♂	0♀ 3♂	6♀ 0♂	15♀ 8♂
*In vitro*	Adults	No	23	23	18	32
		EW (mg)	256.4 ± 102.5	205.3 ± 68.9	195.7 ± 72.0	239.3 ± 92.4
	Nymphs	No	48^no^	87^n^	65	76^o^
		EW (mg)	2.8 ± 0.9	2.8 ± 0.9	2.7 ± 0.9	2.7 ± 0.8
		Molting rate (%)	54^pq^	34	26^p^	29^q^
		Sex of nymphs	10♀ 16♂	8♀ 22♂	5♀ 12♂	7♀ 15♂

*No, number of engorged ticks; EW, engorgement weight. Mean values ± SD tested in a one-way analysis of variance (ANOVA) followed by Kruskal-Wallis test for multi-group comparisons. *P*-values for number of ticks and molting rate (in percent) were tested by mid-p-exact test. *P*-values were adjusted according to Holm correction. Significantly differences are indicated by letters and *p*-values. The sex of nymphs was determined after molting.*

*^*a*^Mid-P-exact test, *p*-value adjustment by Holm correction; *p* < 0.0001. ^*b*^Mid-P-exact test, *p*-value adjustment by Holm correction; *p <* 0.0001. ^*c*^Mid-P-exact test, *p*-value adjustment by Holm correction; *p <* 0.0001. ^*d*^Mid-P-exact test, *p*-value adjustment by Holm correction; *p <* 0.0001. ^*e*^Mid-P-exact test, *p*-value adjustment by Holm correction; *p <* 0.0001. ^*f*^Kruskal-Wallis test, Dunn’s *post hoc*-test; *p <* 0.0001. ^*g*^Kruskal-Wallis test, Dunn’s *post hoc*-test; *p <* 0.0001. ^h^Mid-P-exact test, *p*-value adjustment by Holm correction; *p <* 0.0001. ^*i*^Mid-P-exact test, *p*-value adjustment by Holm correction; *p <* 0.0001. ^*j*^Mid-P-exact test, *p*-value adjustment by Holm correction; *p <* 0.0001. ^*k*^Mid-P-exact test, *p*-value adjustment by Holm correction; *p <* 0.0001. ^*l*^Mid-P-exact test, *p*-value adjustment by Holm correction; *p <* 0.0001. ^*m*^Mid-P-exact test, *p*-value adjustment by Holm correction; *p* = 0.0133. ^*n*^Mid-P-exact test, *p*-value adjustment by Holm correction; *P* = 0.0002. ^*o*^Mid-P-exact test, *p*-value adjustment by Holm correction; *P* = 0.0127. ^*p*^Mid-P-exact test, *p*-value adjustment by Holm correction; *P* = 0.0173. ^*q*^Mid-P-exact test, *p*-value adjustment by Holm correction; *P* = 0.0292.*

### *In vitro* Tick Feeding

For the first study, the percentage of ticks that attached and successfully fed in the ATFS ranged from 33 to 44% per group for females and from 35 to 42% for nymphs. No significant differences in number of ticks that completed feeding and their engorgement weights were found. The attachment and engorgement rates of the second study were comparable to that of the first and ranged from 24 to 44% for both females and nymphs. Slightly higher female engorgement weights compared to that of the first *in vitro* study were recorded. Again, an effect of *in vitro* feeding of either stored plasma or fresh whole blood on tick engorgement was not observed. Mean engorgement weights and statistical results for adult ticks and nymphs of 1st and 2nd immunization study are shown in Tables 1, 2, respectively.

## Discussion

Immunization with recombinant IrFER2 or native tick proteins elicited a clear antibody response in the immunized animals. Interestingly, sera of the animal vaccinated with ME also recognized salivary gland antigens in the indirect ELISA and vice versa (Figure 3), suggesting the presence of common epitopes in both ME and SGE. Similar findings were previously reported in cattle vaccinated with *R. microplus* gut membrane antigens (Opdebeeck and Daly, 1990) and recent data for *I. ricinus* also indicates the presence of shared transcripts and proteins between salivary glands and midguts (Schwarz et al., 2014; Perner et al., 2016). It is not known if these common epitopes are peptides or carbohydrates; efforts to deglycosylate the tissue extracts by enzymatic deglycosylation or sodium hydroxide to investigate this in more detail were not successful (data not shown). It is also striking that the strongest cutaneous response was observed in the ME-vaccinated animals, with a marked papular swelling at the tick attachment site and extensive serous exudate, a finding that supports the presence of shared epitopes between ME and saliva proteins. The regurgitation of midgut proteins during the feeding process might form an alternative explanation for the observed cutaneous response. There is some evidence that ticks may regurgitate gut contents during feeding (Brown, 1988) and it has also been suggested that pathogens might be transmitted by this route (Burgdorfer et al., 1989). However, the presence of a pharyngeal valve in ticks, which is considered to be an effective barrier preventing regurgitation, argues against this, making regurgitation of gut contents a little understood and controversial phenomenon (Sonenshine and Anderson, 2014). The skin reaction became apparent at 48 h after the ticks were placed on the ear, which together with the observed response is indicative of a type IV delayed hypersensitivity (DTH) reaction. Nymphs in particular might have become trapped in the exudate and died as a result, which may have contributed to the low number of ticks recovered from this animal. DTH reactions have also been observed in immunization studies targeting other hematophagous arthropods; immunization with a 15 kD salivary gland protein of the sandfly vector of leishmaniosis, *Phlebotomus papatasi* (Valenzuela et al., 2001) resulted in a humoral and strong DTH responses upon subsequent exposure to *P. papatasi* and immunization with the 64 TRP antigen derived from the cement cone of *R. appendiculatus* ticks was also shown to induce strong humoral and DTH responses (Trimnell et al., 2005).

Although the FER2 immunized calf developed a high antibody titer, tick feeding on this calf was not impaired. This is in contrast to previous findings of a immunization experiment in which the tick number, engorgement weight, oviposition and fertility of *I. ricinus* feeding on immunized rabbits (*n* = 2) were reduced after threefold immunization with 100 μg IrFER2 (Hajdusek et al., 2010). The small group size or use of individual animals in both studies, animal species-specific differences and differences in the immunization schedule might explain these contrasting findings. Recombinant IrFER2 produced in *E. coli* was not recognized by serum from the TPE vaccinated animal. This can be explained by the assumption that the amount of ferritin 2 in the TPE was minute, as it is mainly expressed in the midgut but secreted into the tick plasma, which was the only tissue where the protein could previously be detected by Western Blot (Hajdusek et al., 2009).

Immunization of calves with native TPEs from adult ticks was more successful in inhibiting tick feeding. Immunization with extracts containing midgut proteins (TPE, ME and a combination of both ME and SGE) in particular resulted in a significant reduction in the number of females and nymphs that fed successfully and also in a significant reduction in the engorgement weights of females. As the native proteins were extracted from adult females, the observed effect on nymphs could be explained by the presence of conserved proteins in both nymph and adult ticks. A recent proteomic study indeed showed a considerable overlap between proteins identified in nymphal and adult salivary glands and midguts (Schwarz et al., 2014).

The effect of immunization with soluble midgut proteins prepared from adult ticks on tick feeding has been previously evaluated for a number of metastriate tick species, including *Amblyomma variegatum, Dermacentor andersoni, Hyalomma anatolicum, H. dromedarii, R. annulatus, R. microplus, R. appendiculatus, R. sanguineus* and the soft tick *Ornithodoros erraticus* (Allen and Humphreys, 1979; Jongejan et al., 1989; Wong and Opdebeeck, 1989; Rechav et al., 1992; Kimaro and Opdebeeck, 1994; Szabo and Bechara, 1997; Banerjee et al., 2003; Manzano-Roman et al., 2006; Nikpay and Nabian, 2016). The reported efficacy of immunization of these trials was, however, rarely as high as reported in this study, with reductions in tick numbers ranging from 63% for ticks feeding on the animal immunized with a combination of SGE and ME, to 97% in the ME-immunized animal. This could again be due to the small number of animals used in this study and/or the mechanical disturbance of ticks caused by the serous exudate at the tick feeding site in the ME-vaccinated animal, but it does indicate that the immunization procedure followed was successful and that the development of tick immunity against *I. ricinus* through immunization in cattle is possible. The optimal amount of native TPEs that confers tick immunity in animals is not known; amounts used in previous studies with cattle ranged from three immunizations with 100 μg salivary gland proteins (Nikpay and Nabian, 2016) to one immunization with 200 mg midgut protein followed by three booster immunizations with 150 mg midgut protein (Essuman et al., 1991). Differences in the tick and host species used, as well as differences in the preparation of antigen extracts and the presentation of vaccine efficacy, make it difficult to draw any conclusions on this matter from previous studies. We therefore followed a pragmatic approach by using the maximum amount of proteins that could be extracted from the partially fed females, which turned out to be in the range of ∼6 to ∼12 mg protein.

Feeding of blood from the vaccinated animals *in vitro* using the ATFS showed that the humoral response of the immunized animals alone did not affect tick feeding. It differs in this regard to the mode of action of the Bm86 vaccine, where the *in vitro* feeding of IgG1 antibodies in particular was shown to cause tick gut damage in *R. microplus* (Kemp et al., 1989). Further identification of the tissue extract antigens that were primarily responsible for the observed protective immune responses would be of great interest in the future development of vaccines targeting *I. ricinus* ticks.

Control of *Ixodes* tick infestations in important reproduction hosts such as deer by using the “4-Poster” acaricide dispensing device was shown to significantly reduce nymphal tick densities in the northeastern United States over time (Brei et al., 2009). Immunization of wildlife hosts against ticks could form an alternative means of reducing tick abundance and the risk for acquiring tick-borne diseases, but will depend on the identification of effective antigens and the availability of suitable vaccine delivery systems such as oral immunization or ballistic delivery of vaccines (Sharma and Hinds, 2012).

Although this work was limited by the small number of animals used due to cost constraints, the results nevertheless indicate that tick immunity against *I. ricinus* can be elicited in cattle upon immunization with native TPEs. Future work will focus on the analysis of immunogenic antigens to identify potential tick-protective vaccine candidates.

## Ethics Statement

This study was carried out in accordance with the recommendations of the Landesamt for Gesundheit and Soziales, Berlin, Germany. The protocol was approved by the Landesamt für Gesundheit und Soziales, under registration number G0210/15.

## Author Contributions

SK, JA, JT, JH, and AN conceived and designed the study. OH and PK contributed materials. SK, JC, JA, and AN performed the experiments. OK performed the histopathological analyses. SK and AN analyzed the data and drafted the manuscript. All authors read and approved the final manuscript submitted for publication.

## Conflict of Interest Statement

The authors declare that the research was conducted in the absence of any commercial or financial relationships that could be construed as a potential conflict of interest.

## Footnotes

1 http://www.graphpad.com

2 http://openepi.com
